# Influence of Biodiesel Content on Fluorescence Anisotropy of Undiluted Diesel-Biodiesel Blends Using Anisotropy-Resolved Multidimensional Emission Spectroscopy (ARMES)

**DOI:** 10.1021/acsomega.5c04717

**Published:** 2025-08-20

**Authors:** Fernando R. Conceição, Luiz E. T. Vilela, Ricardo R. F. Bento, Gustavo Nicolodelli, Samuel L. Oliveira, Anderson R. L. Caires

**Affiliations:** † Optics and Photonics Group, Institute of Physics, 54534 Federal University of Mato Grosso do Sul, PO Box 549, 79070-900 Campo Grande, MS, Brazil; ‡ Federal Institute of Mato Grosso do Sul, 79750-000 Nova Andradina, MS, Brazil; § Institute of Physics, Federal University of Mato Grosso, 78060−900 Cuiabá, MT, Brazil; ∥ Department of Physics, Federal University of Santa Catarina, 88040-900 Florianópolis, Santa Catarina, Brazil

## Abstract

The production of diesel-biodiesel blends (DBB) aims to mitigate the environmental impacts of diesel combustion. However, gaps remain in understanding their molecular properties, particularly fluorescence anisotropy (FA), which reflects molecular rotation and environmental constraints (e.g., viscosity, polarity). This property depends on the viscosity and polarity of the medium and understanding it can provide valuable insights for the development of robust analytical methods to evaluate the biodiesel content in undiluted DBB. In this study, anisotropy-resolved multidimensional emission spectroscopy (ARMES) was applied to investigate the FA, providing new insights into the molecular behavior of these mixtures under undiluted conditions. The biodiesel samples were produced through the transesterification of soybean oil, and the DBB mixtures were prepared with varying biodiesel contents (0 to 100% w/w). ARMES was applied to the samples, enabling the acquisition of anisotropy maps (AM) for each mixture. It was observed that anisotropy varied significantly with the biodiesel content and was more pronounced in intermediate blends (B10 to B50) due to the increased viscosity of the samples. Additionally, the polarity of the mixtures influenced molecular rotation, affecting the observed anisotropy values. The ARMES technique proved to be effective in analyzing complex systems such as DBB, providing new insights into the molecular behavior of these mixtures in undiluted environments.

## Graphical abstract

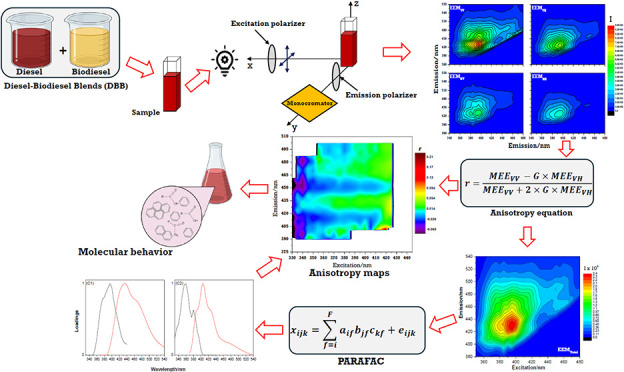

## 1 Introduction

To mitigate the environmental effects caused by diesel combustion, commercial diesel is mixed with biodiesel to form what are known as diesel-biodiesel blends (DBB). Since DBB contains multiple fluorophores in its composition, such as polycyclic aromatic hydrocarbons (PAHs) present in diesel^1,2^ as well as tocopherol, chlorophyll, riboflavin, vitamins (A, K, and D), carotenoids, methyl esters, and oxidation products found in biodiesel.^2–4^ Additionally, the fluorophores present in biodiesel and diesel correlate with key fuel properties such as viscosity, polarity, and oxidative stability, influencing their behavior in fluorescence-based analysis.^5–7^ For instance, in diesel, the dominant fluorophores are the polycyclic aromatic hydrocarbons (PAHs, e.g., pyrene, benzo­[*a*]­pyrene), which are nonpolar and hydrophobic, aligning with low polarity and higher viscosity due to long-chain hydrocarbons. In contrast, biodiesel contains polar fluorophores like tocopherols and oxidation products, reflecting its higher polarity from ester groups and unsaturated fatty acids. Thus, fluorescence spectroscopy (FS) can be applied not only to distinguish biodiesel from diesel but also to assess the composition and molecular properties of DBB.^3,4,8–13^

Several studies based on the FS of DBB have focused on developing analytical methods for quantifying biodiesel content in DBB.^8,10,14^ However, studies investigating the molecular properties of DBB as a function of biodiesel content using FS remain limited.^15^

For the analysis of multifluorophoric systems, such as DBB, multidimensional fluorescence (MF) is the most suitable technique, as it enables the integration of spectral information, including intensity, excitation, and emission wavelengths of the multiple fluorophores, into a single matrix.^16^ This matrix can be obtained through the Excitation–Emission Matrix (EEM) mode and Total Synchronous Fluorescence Spectroscopy (TSFS), each of which has its advantages and disadvantages.^16^ In the EEM mode, the advantage is that the trilinearity of the data is preserved, which facilitates the application of chemometric methods, such as PARAFAC. On the other hand, the presence of Rayleigh and Raman scattering can make it more challenging to obtain models that accurately describe the system under study.^17–19^ Regarding TSFS, it is possible to obtain the total fluorescence of the samples without scattering. However, the application of chemometric methods, such as PARAFAC, requires that the synchronous matrices be converted into trilinear structures before analysis, which requires extra steps in data preprocessing.^20,21^ Thus, the EEM mode requires less data preprocessing, which is why it was adopted in this study.

In addition to containing information about MF, polarized EEM (EEMp) can be obtained to determine the fluorescence anisotropy (FA) (*r*) of the sample.^16,22,23^ *r* measures the polarization of emitted light relative to the excitation light and reflects molecular rotation and environmental constraints. Higher *r* values indicate restricted molecular rotation, which can be promoted by increased viscosity, e.g., for *r* determination, polarizers are added to the excitation and emission monochromators to obtain EEMp in vertical–vertical (VV), vertical–horizontal (VH), horizontal-vertical (HV), and horizontal-horizontal (HH) polarizations in the excitation and emission beams, respectively.^24^ With these matrices, the *r* values of the system can be calculated.^16,22,23,25^ Additionally, chemometric methods such as PARAFAC and MCR are used to resolve overlapping signals among sample constituents, allowing the evaluation of fluorescence anisotropy for individual components.^16,22,23,26^

Fluorescence anisotropy, MF, and chemometrics are elements that, when combined, generate anisotropy-resolved multidimensional emission spectroscopy (ARMES). This technique generates a comprehensive, multidimensional data structure that correlates excitation wavelengths (λ_ex_), emission wavelengths (λ_em_) or Δλ (TSFS), intensity (*I*), and fluorescence anisotropy (*r*). Due to its sensitivity to structural changes, AM, obtained through ARMES, provides additional information about the structure of the molecules that comprise the system under study.^23^ In this context, DBB contains a range of fluorescent molecules, which may exhibit different fluorescence behaviors due to the addition of biodiesel, as biodiesel increases the viscosity,^15^ and polarity^27^ of the DBB. In this context, the present study will, for the first time, introduce the application of ARMES to assess the behaviors of fluorophores comprising DBB in undiluted environments. In this work, soybean biodiesel was selected as the model due to its dominant role in biodiesel production in Brazil. However, the broader relevance of our findings is supported by prior work from our group, which showed that the fluorescence of DBB is feedstock-independent, exhibiting consistent emission profiles and fluorescence intensity across biodiesel derived from soybean, canola, sunflower, and corn oils.^28^

## 2 Materials and Methods

### 2.1 Biodiesel Production and Blend Characterization

Biodiesel was synthesized via base-catalyzed transesterification of refined soybean oil with a 1:6 molar ratio (oil/methanol) using KOH (2% w/w relative to oil mass). The catalyst was dissolved in methanol at 50 °C under stirring and then added to the oil at 60 °C, with the reaction mixture stirred for 2 h to ensure complete conversion. After settling for 24 h in a separatory funnel, two distinct phases formed (Figure S1): the upper phase (biodiesel) and the lower phase (glycerol). The biodiesel phase was washed three times with distilled water to remove glycerol and catalyst residues, followed by two washes with saturated NaCl solution to eliminate residual polar impurities. Finally, the biodiesel was dried over MgSO_4_, filtered, and rotary-evaporated at 60 °C under moderate vacuum (1 h) to remove trace solvents. Fourier transform infrared (FTIR) analysis (Figure S2) confirmed successful transesterification (>95% yield of fatty acid methyl esters, FAMEs), evidenced by the shift in the C=O stretching band from 1746 cm^–1^ (soybean oil) to 1743 cm^–1^ (biodiesel).^29–32^

The DBBs were prepared using the diesel S500 standard marketed by Taurus Petroleum Ltda. To produce blends, a total mass of 15 g was used for each sample, and with biodiesel content increasing in 10% w/w increments from 0 to 100%. The blends are named B*x*, where *x* represents the percentage of biodiesel mass in the blend. The experiments were performed in duplicate, and all experiments were carried out using undiluted samples.^10^ The plastic viscosity (η) of the DBB was measured using a Brookfield LVDV-III digital rheometer with the Bingham model in Rheocalc V2.5 software. A thermostatic bath maintained the temperature at 25 ± 5 °C, and a coaxial-cylinder setup (Spindle SC4–18, 100 mm outer diameter) was used for testing.

### 2.2 Evaluation of the Polarizers

The polarized excitation–emission matrices (EEMp) were acquired after verifying polarizer alignment and optical transmission. Excitation and emission polarizers (Newport RSP-1T, ± 1.0° accuracy) were mounted on rotating bases (Figures S3–S4) and attached to a PerkinElmer Lambda 265 UV–vis spectrometer. Transmittance spectra were recorded using air as a baseline for four polarizer configurations: VV (vertical–vertical), VH (vertical–horizontal), HV (horizontal-vertical), and HH (horizontal-horizontal). Proper alignment was confirmed by (1) high transmittance in VV and HH configurations, and (2) near-zero transmittance in VH and HV configurations.

### 2.3 Optical Characterization

#### 2.3.1 Evaluation of the Transesterification Reaction

FTIR measurements were used to confirm the transesterification of soybean oil. The absorbance spectra of soybean oil and biodiesel were recorded using a PerkinElmer Spectrum 100 series mid-infrared spectrometer. The instrument was equipped with an attenuated total reflectance (ATR) accessory fitted with a germanium crystal. All spectra were collected in the range of 3200–1600 cm^–1^ with a resolution of 1 cm^–1^ and ten scans, at room temperature.

#### 2.3.2 Obtaining the Polarized Excitation–Emission Matrices of Undiluted Diesel-Biodiesel Blends

To obtain the EEMp, the polarizers installed in the excitation and emission channels of the FluoroMate FS-2 spectrofluorometer (SCINCO, Seoul, Korea), which consists of excitation and emission monochromators, a 150 W xenon lamp, and an R-928 photomultiplier as the detector, as shown in Figure S5. A quartz cuvette with four polished faces and optical path lengths of 3 and 10 mm was used as the sample holder. Excitation was performed along the axis corresponding to the shorter optical path of the cuvette. The excitation and emission wavelengths were obtained in the ranges of 330–480 and 360–540 nm, with increments of 5 and 1 nm, respectively. The slit widths for both the excitation and emission monochromators were set to 5.0 nm. All EEMp were collected with the photomultiplier voltage set at 550 V and with an angle of 90° between the excitation and emission beams. Additionally, the integration time, response time, and scan rate were set to 20 ms, 0.1 s, and 600 nm min^–1^, respectively. All EEMp were collected at room temperature without any dilution.

### 2.4 Building Anisotropy Maps

To construct the anisotropy maps (AM), *r* values were calculated according to the following equation^23,24,26^

1 $$r=\frac{{\mathrm{EEM}}_{\mathrm{VV}}-{G\times \mathrm{EEM}}_{\mathrm{VH}}}{{\mathrm{EEM}}_{\mathrm{VV}}+2\times G\times {\mathrm{EEM}}_{\mathrm{VH}}}$$

where EEM_VV_ correspond to the polarized EEM with vertical excitation/emission, EEM_VH_ represents the polarized EEM with vertical excitation/horizontal emission, and *G* is the correction factor for detector sensitivity (eq 2)^23–25^

2 $$G=\frac{{\mathrm{EEM}}_{\mathrm{HV}}}{{\mathrm{EEM}}_{\mathrm{HH}}}$$

For preprocessing of the EEMp, calculating the *G* factor, and determining the *r* values, the software MATLAB R2020a and the EEM package^19^ were used. First, the *G* factor was calculated from the EEM_HV_ and EEM_HH_ of each DBB, without preprocessing, as they did not exhibit scattering in the analyzed region. Next, the data matrices for *G* factor, corresponding to each DBB, were smoothed using the Savitzky-Golay smoothing method. For this, a second-order polynomial and 64 points were adopted as smoothing parameters. Additionally, the polynomial order and the number of points were chosen based on the following criteria: (1) maximum reduction of noise and (2) no alteration of the spectral shape of the EEMMp. After smoothing the matrices of the factor *G*, EEM_VH_ were corrected, and eq 1 can be rewritten as follows

3 $$r=\frac{{\mathrm{EEM}}_{\mathrm{VV}}-{\mathrm{EEM}}_{\mathrm{VH}\prime }}{{\mathrm{EEM}}_{\mathrm{VV}}+2\times {\mathrm{EEM}}_{\mathrm{VH}\prime }}$$

where EEM_VH_′ is the EEM_VH_ matrix corrected by the factor *G*.^23^ Subsequently, the EEM_VV_ and EEM_VH_′ were smoothed to remove any potential noise.^23^ For this, the same method and criteria used for smoothing the matrices of factor *G* were considered. Additionally, the removal of Rayleigh scattering and interpolation was applied to the matrices that exhibited scattering.

After preprocessing the EEMp, the *r* values were calculated using eq 3. Consequently, AM of dimension *r* × λ_ex_ × λ_em_ (anisotropy × excitation wavelength × emission wavelength) were constructed for each DBB.

With the *r* values obtained, the angle between the absorption and emission transition dipoles (β) for the blends between B0 and B100 was calculated as follows

4 $$r=\frac{2}{5}\left(\frac{3\cos^{2}\beta -1}{2}\right)$$

where *r* is the observed anisotropy value.^25^

### 2.5 PARAFAC Model of the EEM_Total_

PARAFAC was applied to the EEM_Total_ matrices (derived from eq 3) to resolve overlapping fluorophore signals and identify optimal anisotropy analysis regions. Data were organized as a three-way array (20 samples × 299 emission × 31 excitation wavelengths), excluding B100 due to structural divergence. Model validation employed: (1) CONCORDIA for component number determination,^33^ and (2) split-half analysis (SHA) to verify robustness.^19^ Decomposition was performed using the EEM toolbox (MATLAB R2020a).^19^

## 3 Results and Discussion

To evaluate the transmission window of the excitation–emission polarizers, their respective transmittance spectra were recorded in the 250–800 nm range, as shown in Figure S6. According to the transmittance spectra obtained in the VV and HH configurations, it can be concluded that the polarizers exhibited a transmission window from 275 to 750 nm. On the other hand, when the polarizers were set to the VH and HV configurations, the transmittance between 275 and 750 nm was close to zero. These results confirm that the excitation–emission polarizers function properly within the 275–750 nm range. Furthermore, based on this information, the positions of the polarizers were defined on the rotary base and adopted as a reference for aligning the polarizers in the VV, VH, HV, and HH polarizations.

Figures S7–S10 present the EEMp of the undiluted DBB, obtained with the excitation–emission polarizers configured in the VV, VH, HV, and HH polarizations, respectively. Emissions in the 380–540 nm range, resulting from excitations between 330 and 480 nm, can be attributed to PAHs and the endogenous fluorophores of biodiesel.^3,29,34–36^ Furthermore, the excitation–emission bands observed in the EEMp of the blends ranging from B60 to B90, in the VV, VH, HV, and HH polarizations, between 340 and 370 nm and 380–440 nm, are related to the oxidation products present in biodiesel and vegetable oils,^4^ such as conjugated dienes, trienes, and tetraenes.^8,37^

In addition, EEM_Total_ of the DBB was obtained from the EEMp and the denominator of eq 3 (Figure 1). EEM_Total_ exhibited the same excitation–emission bands as the EEMp, which are attributed to the PAHs and the endogenous fluorophores of biodiesel.^3,29,34–36^

**Figure 1:**
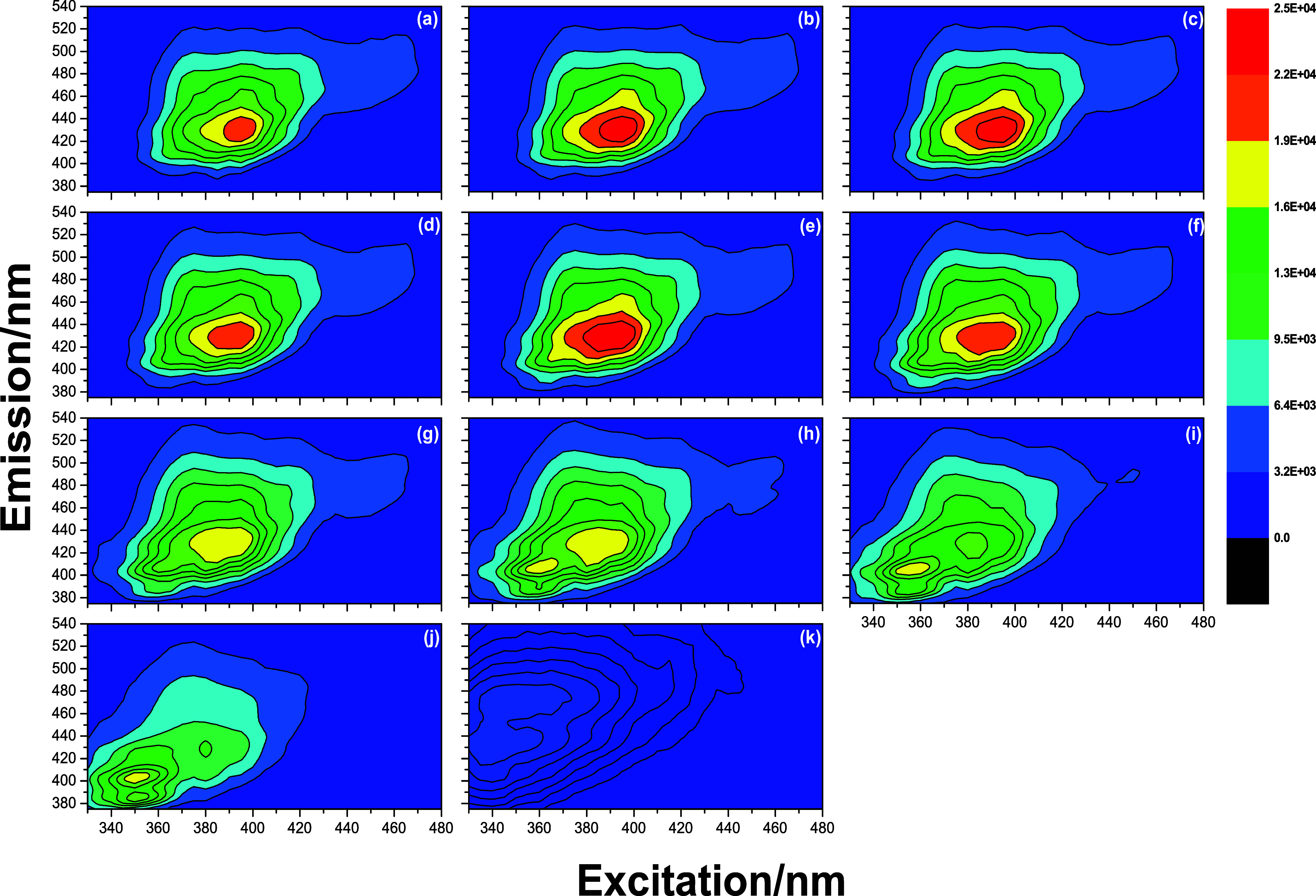
Polarized excitation–emission matrices (EEM_Total_) of the DBB obtained through the denominator of eq 3 for the following blends: (a) B0, (b) B10, (c) B20, (d) B30, (e) B40, (f) B50, (g) B60, (h) B70, (i) B80, (j) B90, and (k) B100.The color scale indicates the calculated emission intensity values.

To extract information about the behavior of these fluorophores in the DBB as a function of biodiesel addition in the blends, the AM was built. Figure 2 shows the AM for each DBB, built from the information contained in the EEMp (Figures S6–S9) and the application of eq 3, where the *x*, *y*, and *z* axes correspond to the excitation wavelengths, emission wavelengths, and the *r* values of the DBB, respectively. In this study, the AM of the DBB was obtained within an emission range between 375 and 510 nm for an excitation range between 330 and 430 nm, with anisotropy values located in the range of 0.21 < *r* < −0.07. The *r* values contained in the AM correspond to the excitation–emission regions of the fluorophores that make up the DBB. Furthermore, the *r* values located in the excitation and emission regions between 400 and 420 nm are not accurate, as this region contains Rayleigh scattering artifacts present in the EEMp in the VV polarization, even after data preprocessing.

**Figure 2:**
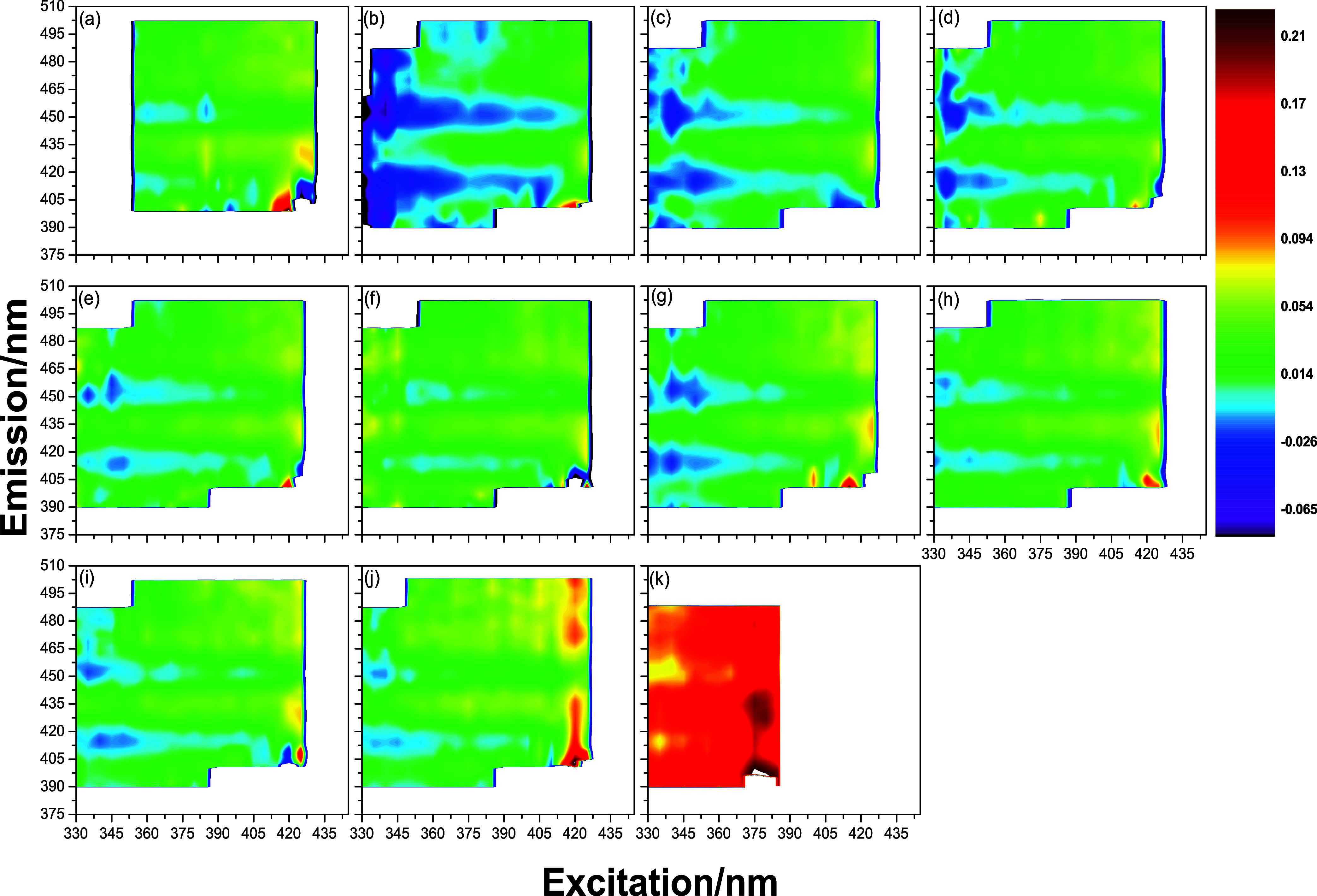
Anisotropy maps (AM) of the blends (a) B0, (b) B10, (c) B20, (d) B30, (e) B40, (f) B50, (g) B60, (h) B70, (i) B80, (j) B90, and (k) B100.

Undiluted DBB is a complex sample that contains various fluorophores contributing to the fluorescence observed in the EEM_Total_. Therefore, identifying the optimal regions where excitations and emissions are located is essential for accurately evaluating the *r* values. Fiona et al. state that ARMES employs PARAFAC and MCR to resolve the overlap between the excitation–emission bands of the fluorophores present in the sample.^16,22,23,26^ Thus, to address the issue of overlap between the excitation–emission bands of the DBB and to obtain the excitation–emission regions of the fluorophore for the analysis of fluorescence anisotropy, PARAFAC was applied to the data set of the EEM_Total_.

The decomposition of the EEM_Total_ using PARAFAC (EEM_Total_- PARAFAC) provided results regarding the excitation and emission spectra of the components (fluorophores) that contributed to the total fluorescence of the DBB. After decomposition, two-component models (C1 and C2) were obtained, with CONCORDIA values of 96.48% (Figure S11). Furthermore, this model was validated by SHA,^19^ with the spectral comparison of the divisions presented in Figure S12. Therefore, these results demonstrate that the EEM_Total_ of the DBB received contributions from two components (C1 and C2).

After the validation of the models, the EEM_Total_ and the bidimensional excitation and emission spectra were retrieved. Figure 3 shows the EEM_Total_ and the retrieved excitation and emission spectra. For component C1 (Figure 3a), the emission between 395 and 535 nm, corresponding to excitation from 355 to 440 nm, was retrieved. As for component C2 (Figure 3b), the retrieved emission occurred between 375 and 510 nm for excitation ranging from 330 to 400 nm. The two-dimensional excitation and emission spectra of components C1 and C2 are presented in Figure 3c,d, respectively. The excitation (emission) wavelengths related to components C1 and C2 showed maximum intensity values centered at 395 nm (435 nm) and 360 nm (405 nm), respectively. Component C1 (λ_ex_/λ_em_ = 395/435 nm) is attributed to polycyclic aromatic hydrocarbons (PAHs) in diesel (e.g., benzo­[*a*]­pyrene), while C2 (λ_ex_/λ_em_ = 360/405 nm) matched endogenous biodiesel fluorophores (e.g., tocopherols).^3,9,38^

**Figure 3:**
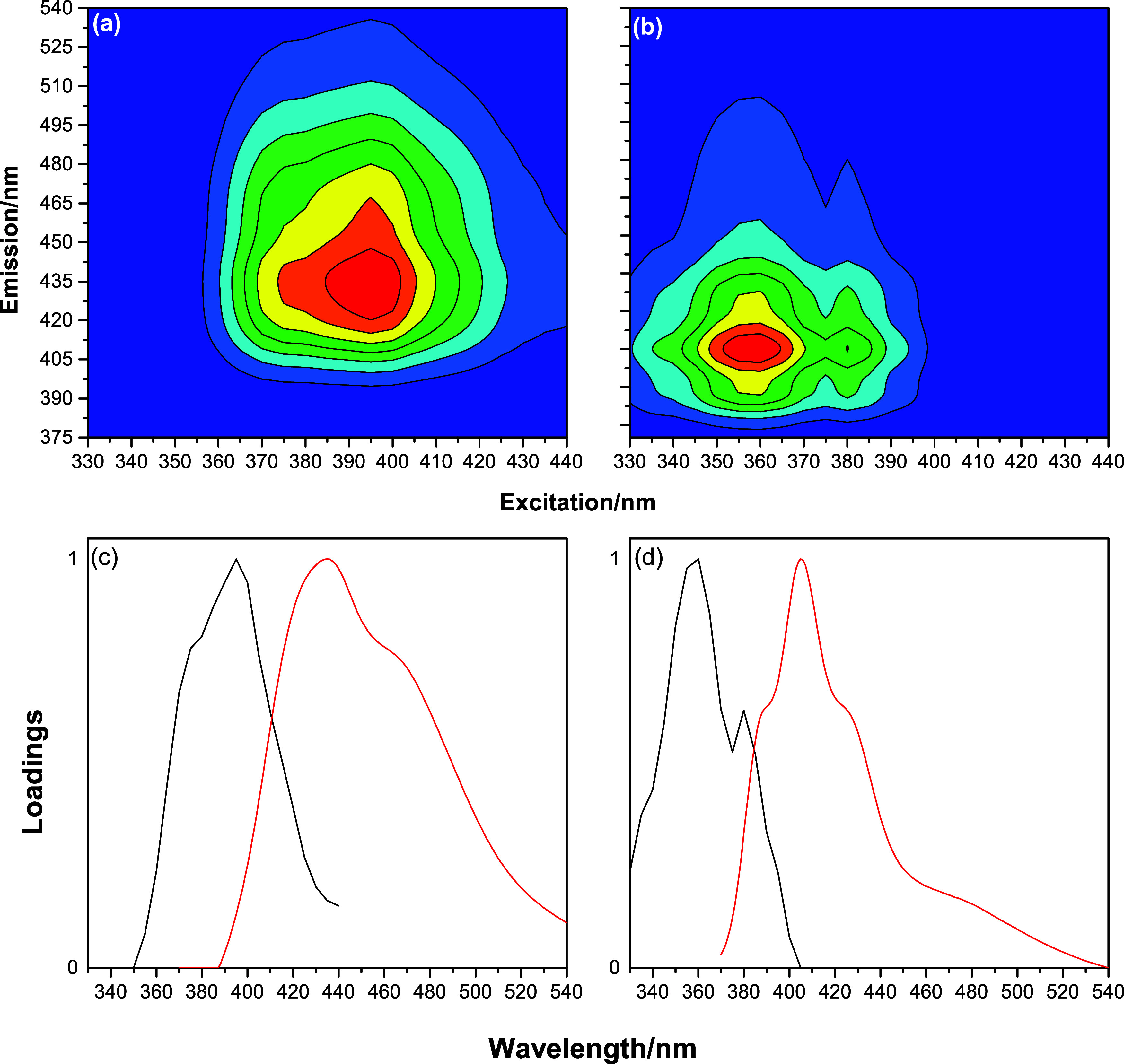
Recovered EEM_Total_ for components (a) C1 and (b) C2, and their respective (c) excitation (black line) and (d) emission (red line).

According to the maximum excitation (emission) wavelengths centered at 395 nm (435 nm) and 360 nm (405 nm), the *r* values were plotted as a function of the biodiesel content, as shown in Figure 4. Significant differences in the *r* values for the B0 and B100 blends were observed, as presented in Figure 4a,4b. To explain this behavior, it is important to consider that diesel molecules (B0) experience a completely different environment compared to biodiesel molecules (B100). Figure S13 shows a linear rise in DBB viscosity with increasing biodiesel content, as expected, due to higher viscosity of biodiesel, from its polar oxygen-containing structure. This result is in accordance with the observed by Caires et al.^15^

**Figure 4:**
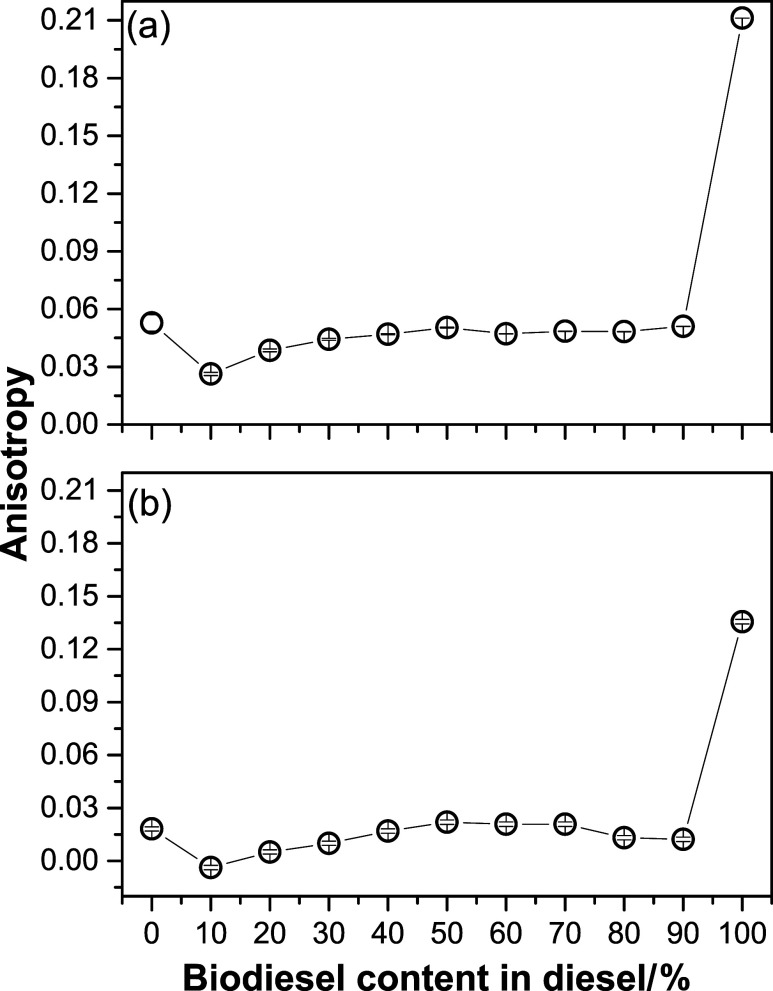
*r* values centered at excitation (emission) wavelengths of (a) 395 nm (430 nm) and (b) 360 nm (405 nm) as a function of biodiesel content in DBB.

With the addition of 10% w/w biodiesel, the *r* value between B0 and B10 decreased, as can be observed in Figure 4a,b. De Souza et al. demonstrated that the dielectric constant of DBB increases with the biodiesel content, which confirms the rise in its polarity.^27^ Consequently, the increase in polarity may cause a reduction in *r* values due to molecular rotation in the excited state,^25,39,40^ explaining the decrease in *r* between B0 and B10.

In addition to polarity, viscosity also plays a fundamental role in the *r* values, which increase as the viscosity of the medium rises.^25^ To evaluate the effect of viscosity on the *r* values, Figure 5 shows the behavior of *r* as a function of the biodiesel content, ranging from B10 to B90. Additionally, the *r* values were obtained from the AM (Figure 2) at the excitation (emission) wavelengths of 395 nm (430 nm) (Figure 5a) and 360 nm (405 nm) (Figure 5b). According to the observed behavior, it was possible to express the infinitesimal variation of fluorescence anisotropy (d*r*) in terms of the infinitesimal variation of biodiesel content (d*B*) in the DBB as follows

5 $$\frac{dr}{dB}=Ce^{-B/K}$$

where *C* is the proportionality constant, *K* is the pre-exponential factor and *B* is biodiesel content in the DBB.

**Figure 5:**
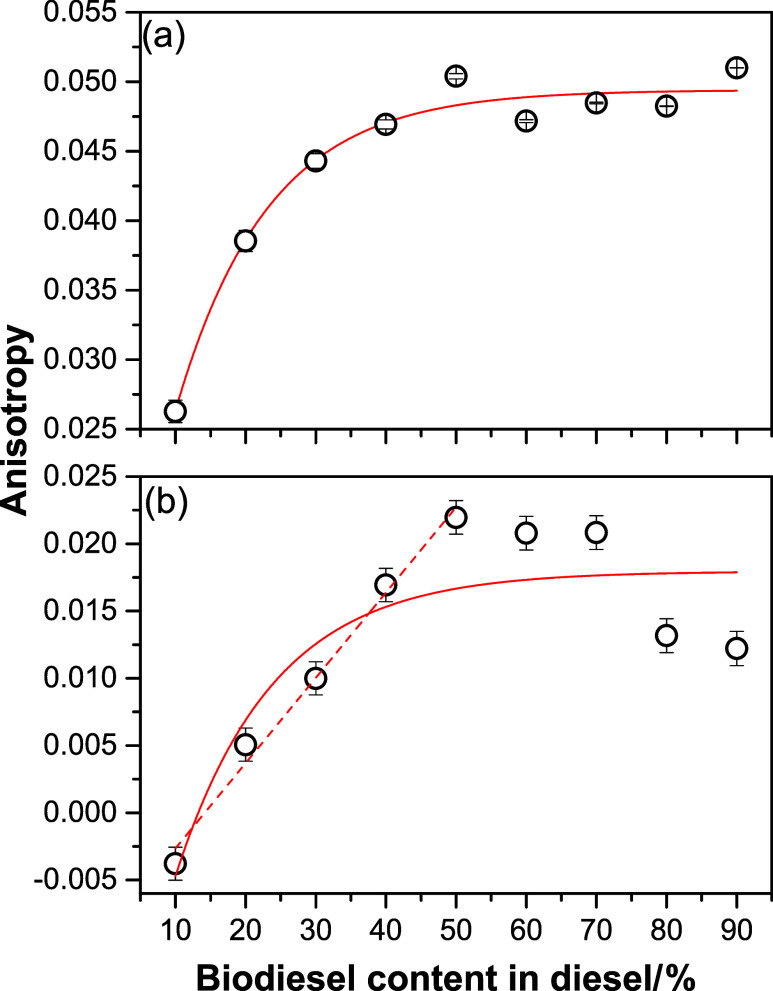
*r* values at the excitation (emission) wavelengths of (a) 395 (435 nm) and (b) 360 (405 nm) as a function of the biodiesel content in diesel within the DBB between B10 and B90. The solid red lines correspond to the adjustments made using eq 7, while the dashed red line represents the linear fit.

Rewriting eq 5, it is possible to obtain the integral that correlates *r* with the biodiesel content in the DBB, as presented below

6 $$\int_{r_{i}}^{r}dr=\int_{B_{i}}^{B}Ce^{-B/K}dB$$

Considering *u* = −*B*/*K* and solving the integral for *u*_*i*_ = 0, it is possible to get a function that correlates *r* with the biodiesel content in the DBB (eq 7)­

7 $$r=A\left(1-e^{-B/K}\right)+r_{i}$$

with *A* = *KC* and *r*_*i*_ equal to the initial anisotropy.

Using eq 7, it was possible to apply it to the data set from Figure 5a,5b, and the equations obtained after fitting were *r*_λ_ex_(λ_em_) = 395(430 nm)_ = 0.050­(1 – e^–*B*/12.96^) and *r*_λ_ex_(λ_em_) = 360(405 nm)_ = 0.046­(1 – e^–*B*/13.94^), with *R* ^2^ = 0.97 e *R* ^2^ = 0.74, respectively. The fit was applied to blends B10 to B90, as *r* values for B0 and B100 deviated from the others. This divergence may be related to the environment in which the fluorophores of B0 and B100 are situated. In other words, the environment is pure and does not exhibit a mixture of diesel and biodiesel, which could alter the conditions for these fluorophores. Regarding the value of *r*_*i*_, its respective values were neglected in the equations *r*_λ_ex_(λ_em_) = 395(430 nm)_ and *r*_λ_ex_(λ_em_) = 360(405 nm)_ because according to the adjustments, their values were approximately zero. Furthermore, a linear fit was applied to the values of *r* obtained at the excitation (emission) wavelengths of 360 (405 nm) between B10 and B50. The equation *r*_λ_ex_(λ_em_) = 360(405 nm)_(B10–B50)__ = (−1 ± 0.1)­10^–2^ + (6.34 ± 0.40)­10^–4^ with *R* ^2^ = 0.99 was obtained, as shown in Figure 5b (dashed line).

Based on the behavior observed in Figure 5, the FA showed an increase up to a limiting value associated with the B50 blend, with *r*_λ_ex_(λ_em_) = 395(435 nm)_ ≈ 0.05 and *r*_λ_ex_(λ_em_) = 360(405 nm)_ ≈ 0.02. The increase in FA values up to B50 may be related to the increase in viscosity in the B50 blends.^25,41^ On the other hand, anisotropy values stabilized around a maximum between B50 and B90 for excitation (emission) at 395 (430 nm) may be related to the minimal mobility of the fluorophores in each blend. In other words, under these conditions, viscosity no longer influences the mobility of the fluorophores, and consequently, the anisotropy values do not increase. Conversely, this same behavior was observed for biodiesel concentrations in diesel between 50 and 70% w/w, during excitation (emission) at 360 nm (405 nm). Furthermore, above 70% w/w, the anisotropy values decreased, which will be explained further on.

It is noteworthy that the values of *r* exhibited an increasing trend with the addition of biodiesel in the DBB. However, these values remained below the maximum anisotropy value. (*r*_0_ ≈ 0.4).^25^ Additionally, *r*_0_ corresponds to the maximum value of *r* in the absence of depolarization between excitation and emission. This condition assumes maximum photoselection of the analyzed fluorophores.^25^ To elucidate this behavior, eq 4 was used to calculate the angle between the transition dipoles of absorption and emission (β), at the excitation (emission) wavelengths of 395 nm (430 nm) and 360 (405 nm). Table 1 presents the calculated values of β for the blends ranging from B0 to B100.

**Table 1:** Values of the Angle between the Transition Dipoles in Absorption and Emission for Each Diesel-Biodiesel Blend (DBB), Obtained at Excitation of 395 nm (Emission at 435 nm) and 360 nm (Emission at 405 nm)

DBB	β 395 (435 nm)	β 360 (405 nm)
0	49.53 ± 0.29	52.92 ± 0.21
10	52.11 ± 0.19	55.12 ± 0.20
20	50.91 ± 0.19	54.22 ± 0.21
30	50.35 ± 0.17	53.73 ± 0.21
40	50.09 ± 0.15	53.04 ± 0.21
50	49.76 ± 0.13	52.54 ± 0.21
60	50.07 ± 0.11	52.65 ± 0.21
70	49.95 ± 0.10	52.65 ± 0.21
80	49.97 ± 0.10	53.41 ± 0.21
90	49.70 ± 0.07	53.51 ± 0.21
100	34.13 ± 0.06	41.59 ± 0.22

Values of *r*_0_ = 0.4 imply that β = 0°, meaning that a highly viscous medium is required. In the case of the blends, the viscosity of the medium increases with the addition of biodiesel. However, the *r* values remain low, with higher β values. This behavior, as previously discussed, may be associated with the increased polarity of the blends due to the addition of biodiesel.^27^ This increase in polarity causes the molecules to depolarize between absorption and emission events, contributing to the rise in β. Moreover, another factor that may contribute to the β values is that their high values (low *r* values) are associated with excitations in higher electronic states, which are generally not the states responsible for emission.^25^ Thus, the electrons in the excited state take some time to reach the state in which emission occurs, allowing the fluorophore to rotate before emitting.^39^ Additionally, the increase in β values and the reduction in *r* values for blends above 70% w/w at the excitation (emission) wavelengths of 360 (405 nm) (Figure 5b) may be related to the increase in the medium’s polarity, which could have influenced the high β values for these blends. Therefore, although the addition of biodiesel increases the viscosity of DBB, this increase was not sufficient to overcome the effects of polarity on the fluorescence anisotropy values of the diluted DBB.

In addition to the influence of excitations in higher electronic states and polarity, the lack of dilution in the blends may also have contributed to the low *r* values, as the solution needs to be diluted to prevent depolarization due to reabsorption and emission, or energy transfer.^25^

## 4 Conclusions

ARMES enabled the construction of the AM for undiluted diesel-biodiesel blends. Using the PARAFAC method, the excitation (emission) regions of the fluorophores comprising the total EEM_Total_ were found to be centered at 395 (435 nm) and 360 (405 nm). The *r* values for each DBB were then obtained through the AM. At these wavelengths, significant differences in *r* values were observed for B0 and B100. This difference was attributed to the medium in which the fluorophores of the B0 and B100 blends are contained, as B100 is more viscous than B0. Additionally, the *r* values decreased between B0 and B10, which was attributed to the increase in blend polarity due to the addition of biodiesel.

An increase in *r* values was observed between the B10 and B50 blends as a function of the biodiesel content in these blends. This increase can be attributed to the rise in viscosity of these blends due to the addition of biodiesel. However, despite the increase in viscosity, low *r* values were found for all blends. These low values were attributed to the effect of polarity on the fluorophores present in the blends, as polarity can cause rotation of molecules in the excited state, leading to an increase in the angle between the excitation and emission transition dipoles (β). In addition to the effects of polarity, variables such as excitation wavelength and the lack of dilution contributed to the low *r* values.

ARMES is a powerful technique for assessing the behavior of fluorophores in their environment. Thus, it was possible for the first time to obtain fluorescence anisotropy values for the fluorophores that comprise the undiluted diesel-biodiesel blends.

## Supplementary Material



## Notes

Image of decantation funnel showing the phases of the biodiesel production process (Figure S1); FTIR spectra of soybean oil and biodiesel samples (Figure S2); images of experimental setup of excitation and emission polarizers on a rotating base, excitation polarizers and emission polarizers added and aligned in the UV–vis spectrophotometer, and configuration of excitation (Ex) and emission (Em) polarizers in the fluorometer (Figures S3, S4, and S5); transmittance spectra obtained with the polarizers adjusted in the following orientations: VV, HH, VH, and HV (Figure S6); EEMp of the blends obtained under VV, VH, HV, and HH polarizations (Figures S7, S8, S9, and S10); CONCONDIA versus number of components (Figure S11); validation of the PARAFAC model through SHA (Figure S12); and viscosity as a function of biodiesel content in DBB (Figure S13) (PDF)

## References

[1] de
Souza C. V., Corrêa S. M. (2016). Polycyclic Aromatic Hydrocarbons
in Diesel Emission, Diesel Fuel and Lubricant Oil. Fuel.

[2] Tomazzoni G., Meira M., Quintella C. M., Zagonel G. F., Costa B. J., de Oliveira P. R., Pepe I. M., da Costa Neto P. R. (2014). Identification
of Vegetable Oil or Biodiesel Added to Diesel Using Fluorescence Spectroscopy
and Principal Component Analysis. J. Am. Oil
Chem. Soc..

[3] Magalhães K., Caires A. R. L., Silva M. S., Alcantara G. B., Oliveira S. L. (2014). Endogenous Fluorescence of Biodiesel and Products Thereof:
Investigation of the Molecules Responsible for This Effect. Fuel.

[4] Kongbonga Y. G. M., Ghalila H., Onana M. B., Majdi Y., Lakhdar Z. B., Mezlini H., Sevestre-Ghalila S. (2011). Characterization of Vegetable Oils
by Fluorescence Spectroscopy. Food Nutr. Sci..

[5] Knothe G., Steidley K. R. (2005). Kinematic Viscosity
of Biodiesel Fuel Components and
Related Compounds. Influence of Compound Structure and Comparison
to Petrodiesel Fuel Components. Fuel.

[6] Yilmaz N., Davis S. M. (2016). Polycyclic Aromatic Hydrocarbon (PAH) Formation in
a Diesel Engine Fueled with Diesel, Biodiesel and Biodiesel/n-Butanol
Blends. Fuel.

[7] Caires A. R. L., Scherer M. D., De Souza J. E., Oliveira S. L., M’Peko J. C. (2014). The Role
of Viscosity in the Fluorescence Behavior of the Diesel/Biodiesel
Blends. Renewable Energy.

[8] Magalhães, K. F. ; Caires, A. R. L. ; Chimenez, T. A. ; Fripp, M. C. ; Machado, F. ; Oliveira, S. L. Fluorescence Spectroscopy as an Alternative Analytical Tool for Monitoring Biodiesel Oxidative Stability: Thermal Oxidation Effect on the Endogenous Chromophores and Fluorophores in Biodiesel. In Green Energy and Technology; Springer, 2018; pp 111–125.

[9] Magalhães K. F., Orellana G., Caires A. R. L., Oliveira S. L. (2023). Fluorescence Lifetime
of Endogenous Fluorophores for Assessing the Thermal Degradation of
Biodiesel. J. Cleaner Prod..

[10] Conceição F. R., Michels F. S., Falcão E. A., Nicolodelli G., Oliveira S. L., Caires A. R. L. (2024). A Fluorescence-based
Multivariate
Method for Biodiesel Quantification in Undiluted Diesel-Biodiesel
Blends without Sample Preparation. Spectrochim.
Acta, Part A.

[11] Alostaz M., Biggar K., Donahue R., Hall G. (2008). Petroleum Contamination
Characterization and Quantification Using Fluorescence Emission-Excitation
Matrices (EEMs) and Parallel Factor Analysis (PARAFAC). J. Environ. Eng. Sci..

[12] Alostaz M., Biggar K., Donahue R., Hall G. (2008). Soil Type Effects on
Petroleum Contamination Characterization Using Ultraviolet Induced
Fluorescence Excitation-Emission Matrices (EEMs) and Parallel Factor
Analysis (PARAFAC). J. Environ. Eng. Sci..

[13] Steffens J., Landulfo E., Courrol L. C., Guardani R. (2011). Application of Fluorescence
to the Study of Crude Petroleum. J. Fluoresc..

[14] Scherer M. D., Oliveira S. L., Lima S. M., Andrade L. H. C., Caires A. R. L. (2011). Determination
of the Biodiesel Content in Diesel/Biodiesel Blends: A Method Based
on Fluorescence Spectroscopy. J. Fluoresc..

[15] Caires A. R. L., Scherer M. D., De Souza J. E., Oliveira S. L., M’Peko J.-C. (2014). The Role
of Viscosity in the Fluorescence Behavior of the Diesel/Biodiesel
Blends. Renewable Energy.

[16] Fiona, G. Anisotropy Resolved Multidimensional Emission Spectroscopy (ARMES) as a Tool for Biophysical Analysis. 2021.

[17] Bro R. (1997). PARAFAC. Tutorial
and Applications. Chemom. Intell. Lab. Syst..

[18] Murphy K. R., Stedmon C. A., Graeber D., Bro R. (2013). Fluorescence Spectroscopy
and Multi-Way Techniques. PARAFAC. Anal. Methods.

[19] Decomposition routines for Emission-Excitation Matrices. https://dreem.openfluor.org/.

[20] Kumar K., Mishra A. K. (2015). Parallel Factor
(PARAFAC) Analysis on Total Synchronous
Fluorescence Spectroscopy (TSFS) Data Sets in Excitation–Emission
Matrix Fluorescence (EEMF) Layout: Certain Practical Aspects. Chemom. Intell. Lab. Syst..

[21] Kumar K., Mishra A. K. (2012). Application of Parallel
Factor Analysis to Total Synchronous
Fluorescence Spectrum of Dilute Multifluorophoric Solutions: Addressing
the Issue of Lack of Trilinearity in Total Synchronous Fluorescence
Data Set. Anal. Chim. Acta.

[22] Casamayou-Boucau Y., Ryder A. G. (2017). Extended Wavelength
Anisotropy Resolved Multidimensional
Emission Spectroscopy (ARMES) Measurements: Better Filters, Validation
Standards, and Rayleigh Scatter Removal Methods. Methods Appl. Fluoresc..

[23] Steiner-Browne M., Elcoroaristizabal S., Casamayou-Boucau Y., Ryder A. G. (2019). Investigating Native
State Fluorescence Emission of Immunoglobulin G Using Polarized Excitation
Emission Matrix (PEEM) Spectroscopy and PARAFAC. Chemom. Intell. Lab. Syst..

[24] Ameloot M., VandeVen M., Acuña A. U., Valeur B. (2012). Fluorescence Anisotropy
Measurements in Solution: Methods and Reference Materials (IUPAC Technical
Report). Pure Appl. Chem..

[25] Lakowicz, J. R. Principles of Fluorescence Spectroscopy; Springer US: Boston, MA, 2006.

[26] Casamayou-Boucau Y., Ryder A. G. (2018). Accurate Anisotropy Recovery from
Fluorophore Mixtures
Using Multivariate Curve Resolution (MCR). Anal.
Chim. Acta.

[27] De
Souza J. E., Scherer M. D., Cáceres J. A. S., Caires A. R. L., M’Peko J.-C. (2013). A Close Dielectric Spectroscopic
Analysis of Diesel/Biodiesel Blends and Potential Dielectric Approaches
for Biodiesel Content Assessment. Fuel.

[28] Caires A. R. L., Lima V. S., Oliveira S. L. (2012). Quantification
of Biodiesel Content
in Diesel/biodiesel Blends by Fluorescence Spectroscopy: Evaluation
of the Dependence on Biodiesel Feedstock. Renewable
Energy.

[29] Chimenez T. A., Magalhães K. F., Caires A. R. L., Oliveira S. L. (2012). Fluorescence as
an Analytical Tool for Assessing the Conversion of Oil into Biodiesel. J. Fluoresc..

[30] Conceição F. R., Michels F. S., Falcão E. A., Nicolodelli G., Oliveira S. L., Caires A. R. L. (2024). A Fluorescence-Based
Multivariate
Method for Biodiesel Quantification in Undiluted Diesel-Biodiesel
Blends without Sample Preparation. Spectrochim.
Acta, Part A.

[31] de
Matos T. S., dos Santos R. C., de Souza C. G., de Carvalho R. C., de Andrade D. F., D’ávila L. A. (2019). Determination of
the Biodiesel Content on Biodiesel/Diesel Blends and Their Adulteration
with Vegetable Oil by High-Performance Liquid Chromatography. Energy Fuels.

[32] Valente V. S. B., dos Santos Vieira A., Teixeira R. M., Rey N. A. (2016). Physicochemical
Characterization of Commercial Biodiesel/Diesel Blends and Evaluation
of Unconventional Spectroscopic Vibrational Techniques in the Monitoring
of Their Oxidation and Hydrolysis during Storage. Energy Fuels.

[33] Bro R., Kiers H. A. L. (2003). A New Efficient
Method for Determining the Number of
Components in PARAFAC Models. J. Chemom..

[34] Caires A.
R. L., Lima V. S., Oliveira S. L. (2012). Quantification of Biodiesel Content
in Diesel/Biodiesel Blends by Fluorescence Spectroscopy: Evaluation
of the Dependence on Biodiesel Feedstock. Renewable
Energy.

[35] Patra D., Mishra A. (2002). Total Synchronous Fluorescence Scan Spectra of Petroleum
Products. Anal. Bioanal. Chem..

[36] Patra D., Lakshmi S. K., Mishra A. (2001). Characterization and Investigation
of Polycyclic Aromatic Compounds Present in Petrol, Diesel, Kerosene
and 2T Oil Using Excitation Emission Matrix Fluorescence. Indian J. Chem., Sect. A.

[37] Insausti M., de Araújo Gomes A., Camiña J. M., de Araújo M. C.
U., Band B. S. F. (2017). Fluorescent
Fingerprints
of Edible Oils and Biodiesel by Means Total Synchronous Fluorescence
and Tucker3Modeling. Spectrochim. Acta, Part
A.

[38] Ferretto N., Tedetti M., Guigue C., Mounier S., Redon R., Goutx M. (2014). Identification and
Quantification of Known Polycyclic Aromatic Hydrocarbons
and Pesticides in Complex Mixtures Using Fluorescence Excitation–Emission
Matrices and Parallel Factor Analysis. Chemosphere.

[39] Haidekker M. A., Brady T. P., Lichlyter D., Theodorakis E. A. (2005). Effects
of Solvent Polarity and Solvent Viscosity on the Fluorescent Properties
of Molecular Rotors and Related Probes. Bioorg
Chem..

[40] Haidekker M. A., Theodorakis E. A. (2007). Molecular Rotors—Fluorescent
Biosensors for
Viscosity and Flow. Org. Biomol. Chem..

[41] Levitt J.
A., Chung P., Kuimova M. K., Yahioglu G., Wang Y., Qu J., Suhling K. (2011). Fluorescence Anisotropy of Molecular Rotors. ChemPhysChem.

