# Supplementary material for: Influence of Biodiesel Content on Fluorescence Anisotropy of Undiluted Diesel-Biodiesel Blends Using Anisotropy-Resolved Multidimensional Emission Spectroscopy (ARMES)
Source: ACS Omega. 2025 Aug 20;10(34):38902–10. doi: 10.1021/acsomega.5c04717 (PMC12409574; doi:10.1021/acsomega.5c04717)
Supplement: Supplementary file 1 [file ao5c04717_si_001.pdf]

Influence of biodiesel content on fluorescence anisotropy of undiluted diesel-biodiesel blends using anisotropy-resolved multidimensional emission spectroscopy (ARMES)

Fernando R. Conceição,[a,b] Luiz E. T. Vilela,[a] Ricardo R. F. Bento,[c] Gustavo Nicolodelli,[d] Samuel L. Oliveira,[a] Anderson R. L. Caires,[a,*]

[a] Optics and Photonics Group, Institute of Physics, Federal University of Mato Grosso do Sul, PO Box 549, 79070-900. Campo Grande, MS, Brazil.

[b] Federal Institute of Mato Grosso do Sul, 79750-000. Nova Andradina, MS, Brazil.

[c] Institute of Physics, Federal University of Mato Grosso, 78060–900. Cuiabá, MT, Brazil.

[d] Department of Physics, Federal University of Santa Catarina, Florianópolis, Santa Catarina, Brazil.

* Corresponding author: anderson.caires@ufms.br

S1 Materials and Methods

S1.1 Transesterification of soybean oil

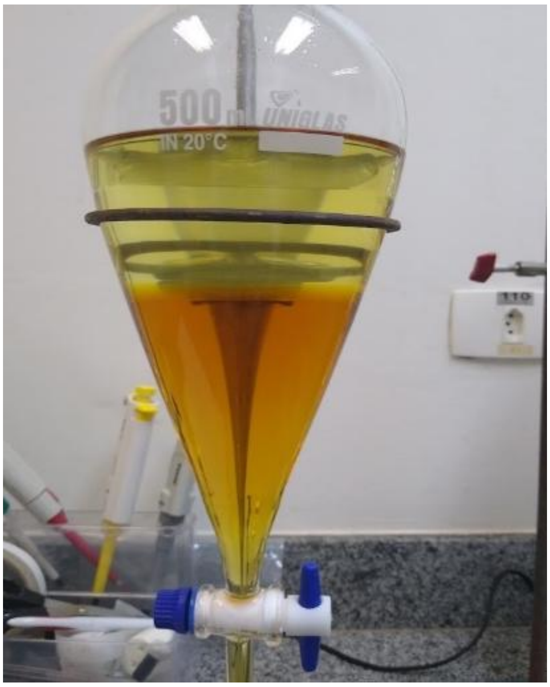

Figure S1. Decantation funnel showing the phases of the biodiesel production process. The biodiesel is the upper phase, while the residues are the lower phase.

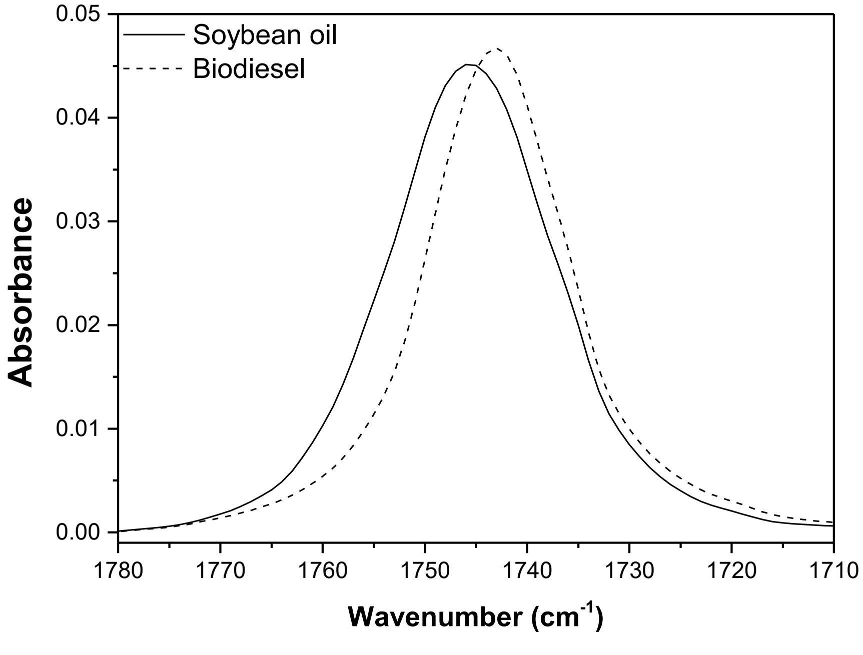

Figure S2. FTIR spectra of soybean oil and biodiesel samples.

S1.2 Evaluation of the Polarizers

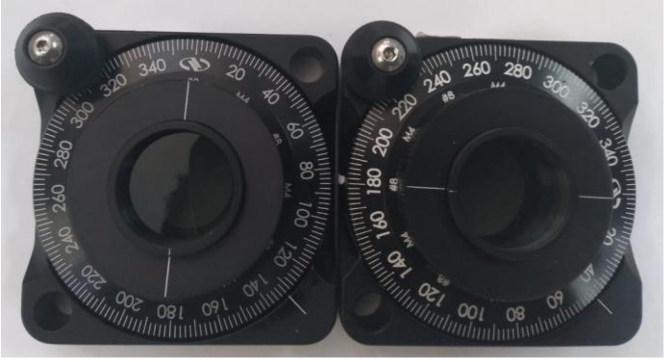

Figure S3. Excitation (left) and emission (right) polarizers on a rotating base.

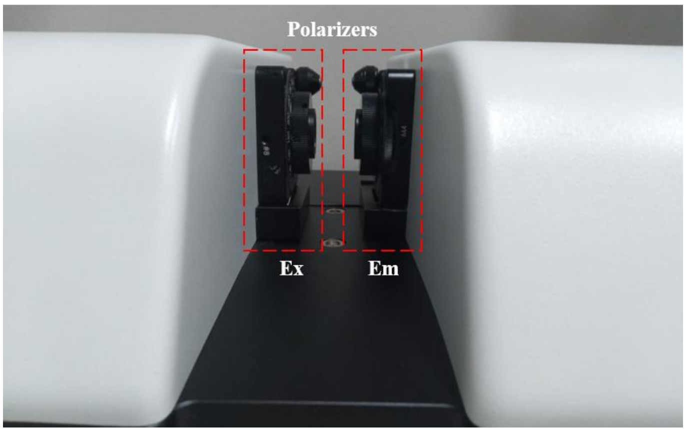

Figure S4. Excitation polarizers (left) and emission polarizers (direct) added and aligned in the UV-Vis spectrophotometer.

S1.3 Optical Characterization - obtaining the polarized excitation-emission matrices of undiluted diesel-biodiesel blends

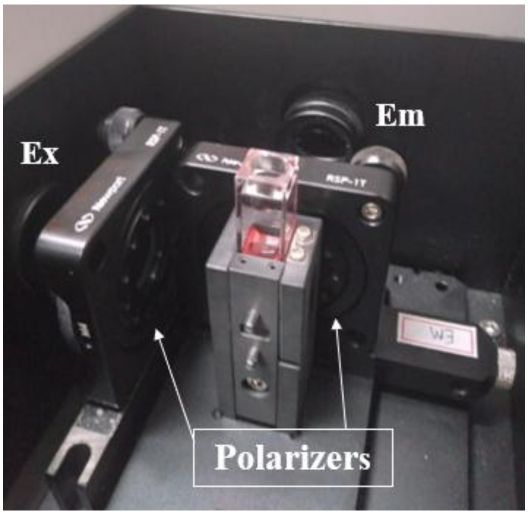

Figure S5. Configuration of excitation (Ex) and emission (Em) polarizers in the fluorometer.

S2 Results and Discussion

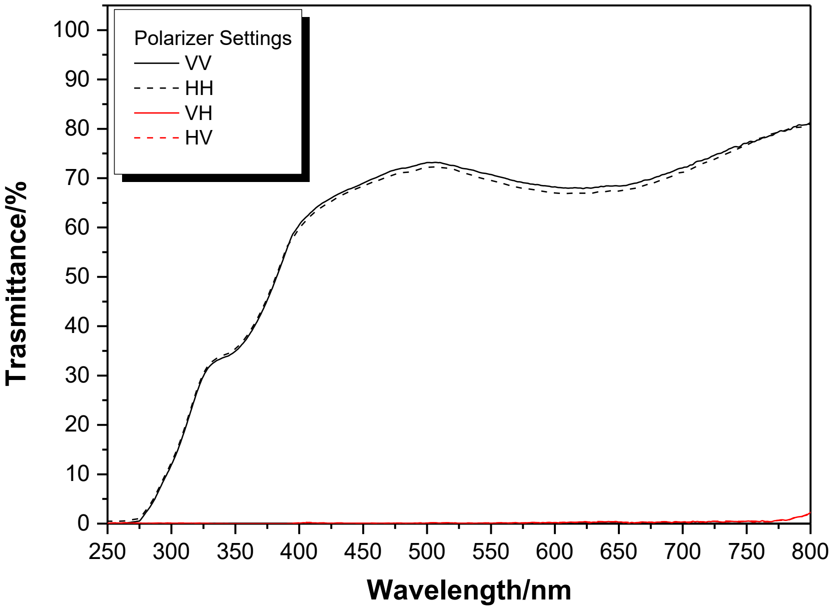

Figure S6. Transmittance spectra obtained with the polarizers set to the following orientations: VV (solid black line), HH (dashed black line), VH (solid red line), and HV (dashed red line).

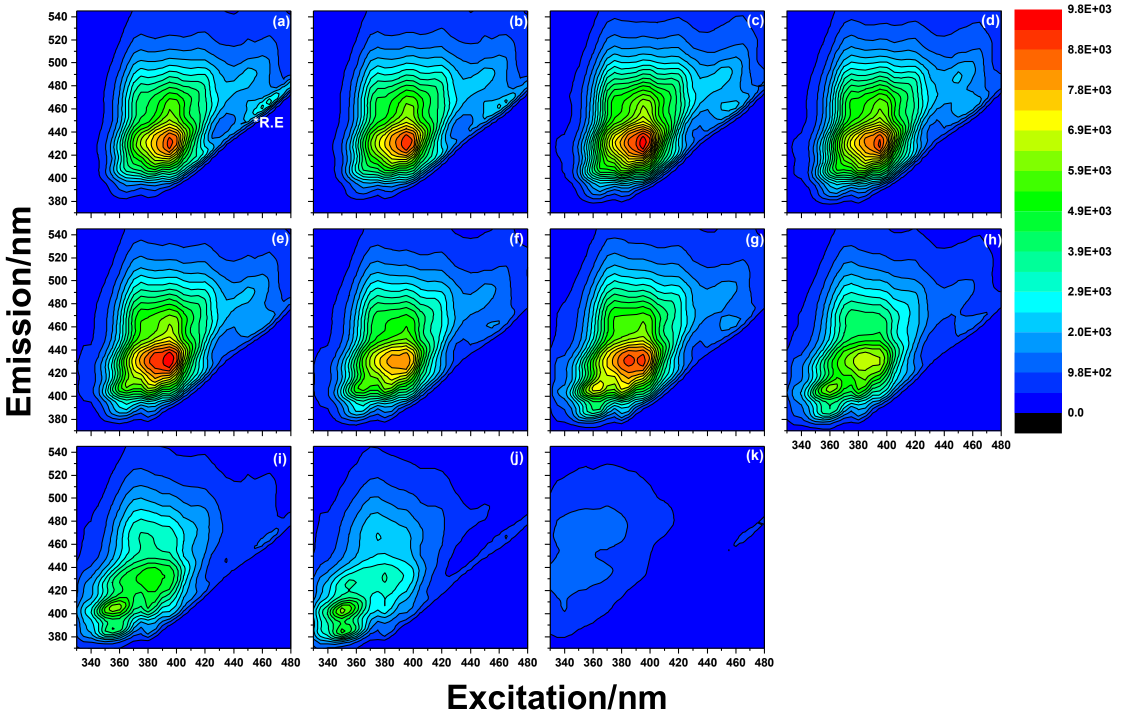

Figure S7. EEMp of the blends: (a) B0, (b) B10, (c) B20, (d) B30, (e) B40, (f) B50, (g) B60, (h) B70, (i) B80, (j) B90, and (k) B100, obtained under VV polarization. The color bar indicates the intensity of polarized fluorescence. R.E. represents Rayleigh scattering.

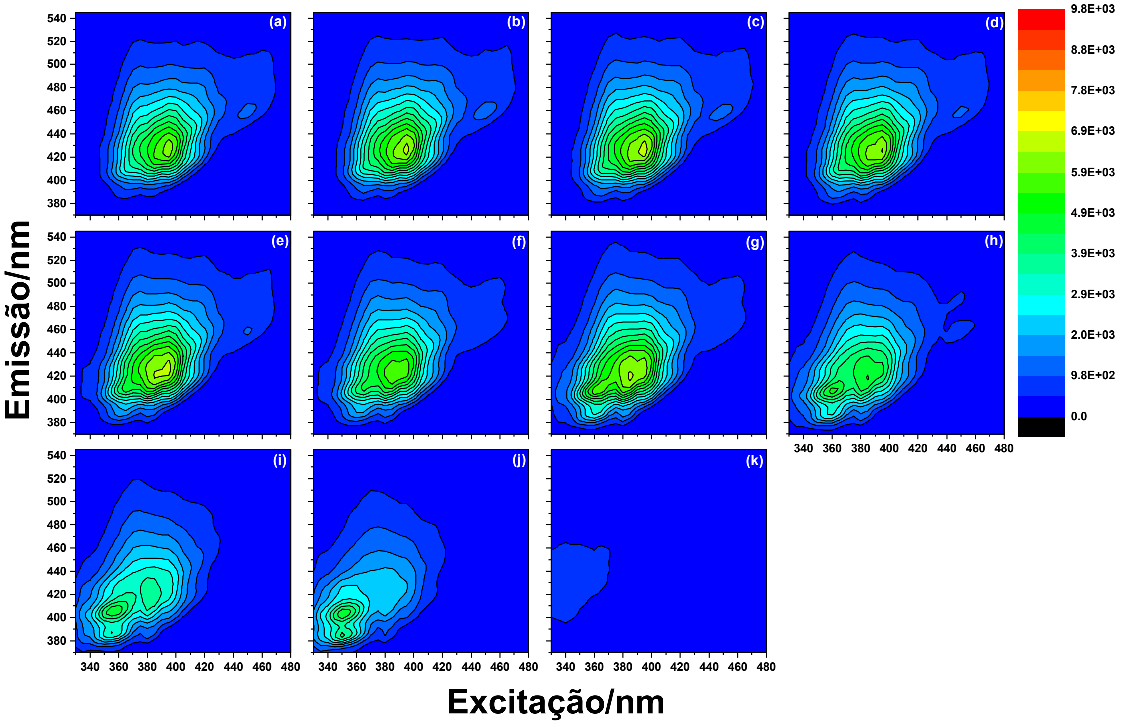

Figure S8. EEMp of the blends: (a) B0, (b) B10, (c) B20, (d) B30, (e) B40, (f) B50, (g) B60, (h) B70, (i) B80, (j) B90, and (k) B100, obtained under VH polarization. The color bar indicates the intensity of polarized fluorescence.

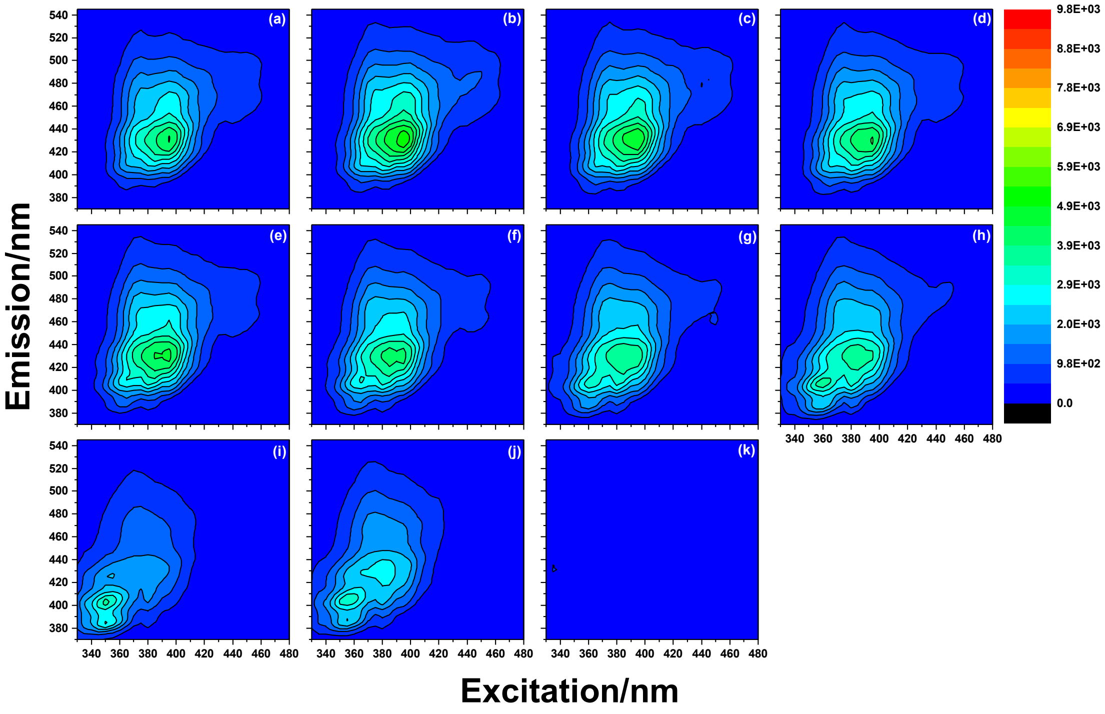

Figure S9. EEMp of the blends: (a) B0, (b) B10, (c) B20, (d) B30, (e) B40, (f) B50, (g) B60, (h) B70, (i) B80, (j) B90, and (k) B100, obtained under HV polarization. The color bar indicates the intensity of polarized fluorescence.

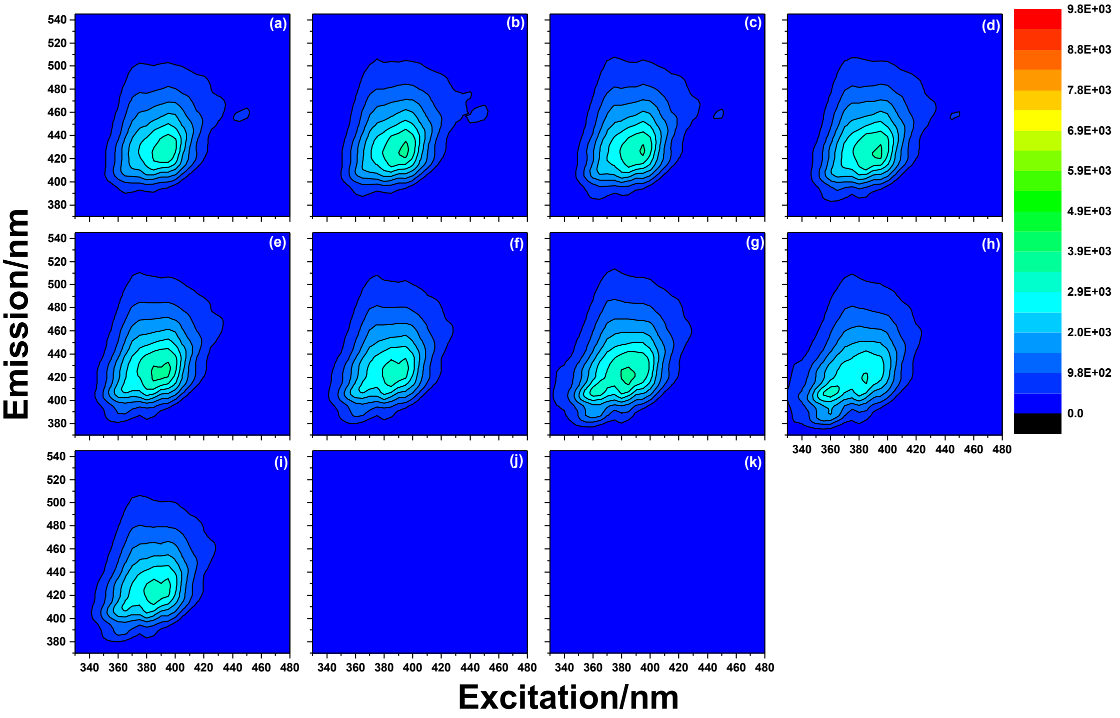

Figure S10. EEMp of the blends: (a) B0, (b) B10, (c) B20, (d) B30, (e) B40, (f) B50, (g) B60, (h) B70, (i) B80, (j) B90, and (k) B100, obtained under HH polarization. The color bar indicates the intensity of polarized fluorescence.

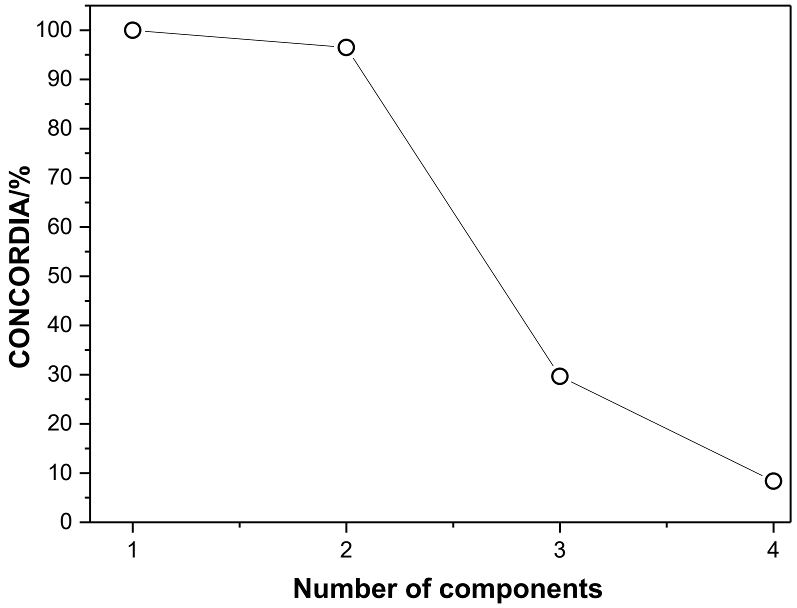

Figure S11. CONCONDIA versus number of components.

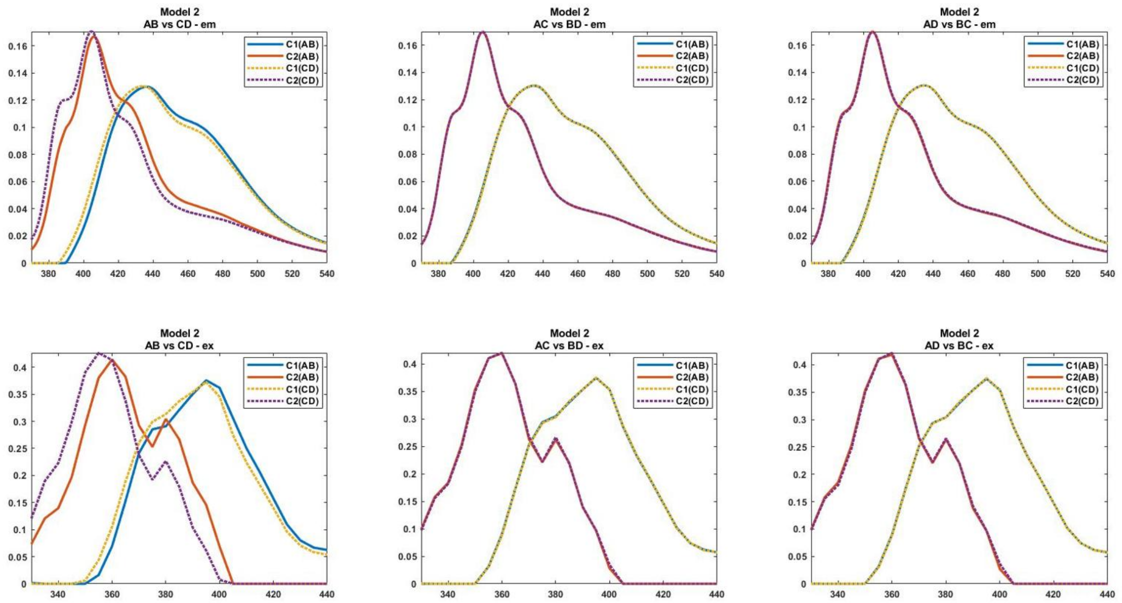

Figure S12. Validation of the PARAFAC model through SHA.

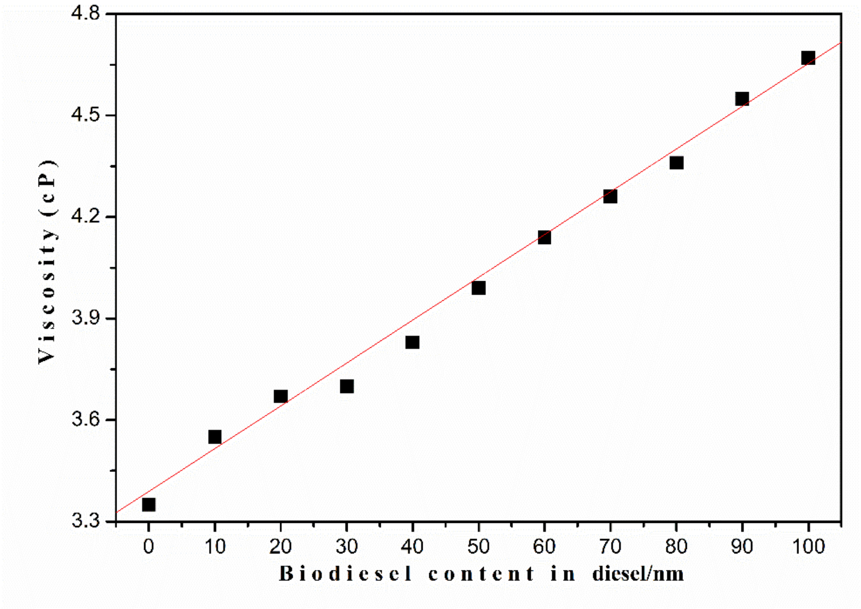

Figure S13. Viscosity as a function of biodiesel content in DBB.
